# Aqueous Synthesis and Characterization of TGA-capped CdSe Quantum Dots at Freezing Temperature

**DOI:** 10.3390/molecules17078430

**Published:** 2012-07-11

**Authors:** Qizhuang Sun, Shasha Fu, Tingmei Dong, Shuxian Liu, Chaobiao Huang

**Affiliations:** College of Chemistry and Life Science, Zhejiang Normal University, Jinhua 321004, Zhejiang, China

**Keywords:** CdSe QDs, thioglycolic acid, freezing temperature

## Abstract

CdSe quantum dots (QDs) have traditionally been synthesized in organic phase and then transferred to aqueous solution by functionalizing their surface with silica, polymers, short-chain thiol ligands, or phospholipid micelles. However, a drastic increase in the hydrodynamic size and biotoxicity of QDs may hinder their biomedical applications. In this paper, the TGA-capped CdSe QDs are directly synthesized in aqueous phase at freezing temperature, and they prove to possess high QY (up to 14%).

## 1. Introduction

In the past twenty years, ®-® semiconductor nanoparticles (so called “quantum dots” or “QDs”) have attracted considerable attention because of their unique optical properties [1,2,3,4]. Among these, colloidal CdSe nanoparticles are among the most widely investigated semiconductor nanoparticles mainly due to their well-tuned emission process and relatively mature synthetic approach [5,6]. Up to now, numerous methods have been reported for the preparation of CdSe nanoparticles, including molecular beam epitaxy [7], metal organic vapor chemical deposition [8], solvothermal [9] and hydro- thermal methods [10]. However, there are only a few methods that can be utilized to synthesize CdSe nanocrystals with satisfactory size and size distributions. For example, an organometallic approach has been widely adopted to prepare CdSe colloid nanocrystals with high quality morphology, high quantum yields as well as good optical quality. However, to inhibit the growth of the particles, a high temperature (200–350 °C) is usually employed in the annealing process, which makes the synthetic procedure for the preparation of CdSe nanocrystals uncontrollable [11,12].

Recently, water-based synthesis of semiconductor nanoparticles with thiols as capping ligands has provided an interesting and alternative method to prepare semiconductor nanoparticles [13]. Compared with the organic system, the aqueous approach is environmentally benign and very economic. Along this line, Pan *et al*. developed an excellent method for the synthesis of high quality CdSe quantum dots under mild reaction conditions, by the use of a two phase water-toluene system [14]. Despite this success, this method suffers from the narrow range of controllable fluorescence owing to the limited Cd precursors [11]. Therefore, it is highly desirable to develop efficient as well as operationally simple methods allowing access to CdSe quantum dots with high quality and controllable fluorescence under mild conditions.

Herein we report an effective synthesis of CdSe quantum dots based on a freezing temperature method. For this synthesis process, CdCl_2_ and Se power are chosen as precursors, and thioglycolic acid (TGA) is used as the capping ligand. Compared to the hot injection and room-temperature injection process, the freezing temperature injection method offers a longer time for the nuclei reactions, and more importantly, the growth of the CdSe crystals could be easily controlled [15].

## 2. Results and Discussion

**Figure 1:**
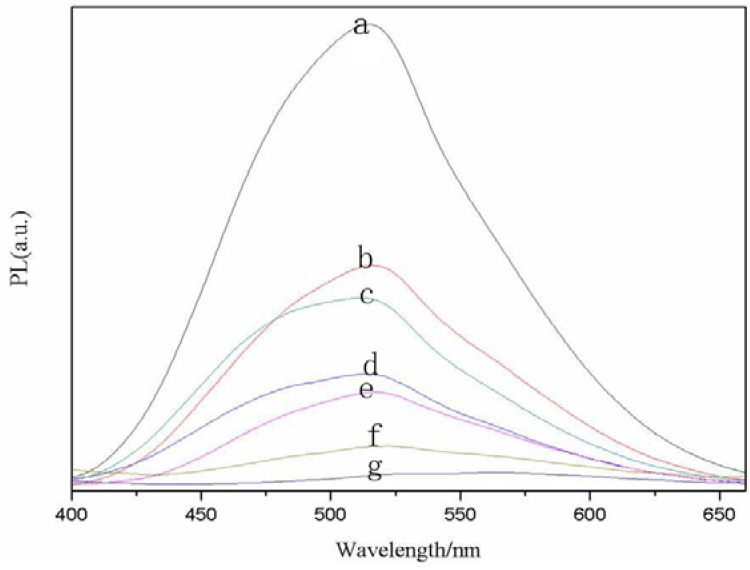
PL spectra of the CdSe quantum dots at different temperatures (a, 0 °C; b, 10 °C; c, 20 °C; d, 40 °C; e, 60 °C; f, 80 °C; g, 100 °C).

Initially, we investigated the influence of reaction temperature on CdSe quantum dot fluorescence intensity. As illustrated in Figure 1, the highest fluorescence intensity of the CdSe QDs is obtained at the freezing temperature, while that of CdSe QDs synthesized at room temperature clearly decreases. 

The increase of molecular kinetic energy at higher temperature will boost the collisions between molecules, thus resulting in more excited state fluorescent molecules through the collision between the molecules or energy transfer between molecules. Non-fluorescence emission forms and leads to quenched fluorescence and lower quantum yield as well as weaker fluorescence intensity. Figure 1 also illustrates that the half peak width of emission spectrum becomes broader with increasing reaction temperature, indicating that the QD size distribution gradually become wider, which can be explained by the Ostwald maturation mechanism. The higher reaction temperature results in a faster monomer deposition. Furthermore, owing to the lower reaction temperature, the lower reaction speed and the higher free monomer concentration of mixtures, the monomer sedimentation processes need more time, and form nanoclusters in the earlier reaction, so the wide emission related to defects will prolong the duration, which results in size distribution attains minimum value.

**Figure 2:**
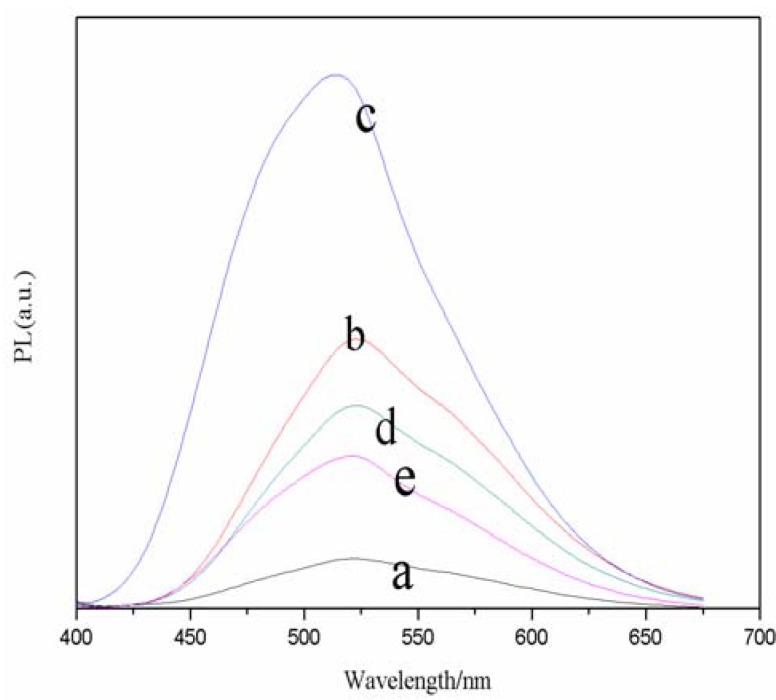
Effects of TGA/Se molar ratios on normalized FL spectra of TGA-capped CdSe QDs at freezing temperature. The reaction time was 30 min (a, 2.0; b, 2.1; c, 2.2; d, 2.3; e, 2.6).

Capping ligands attached to the surface of nanocrystals are found to be important to the formation of the nanocrystals. They can not only prevent the aggregation of small crystals, but also influence the morphology of the nanocrystals because of their different complexation to the crystal. As such, to study the effect of molar ratio on fluorescence intensity, the molar ratio of TGA/Se was varied from 2.0 to 2.6 while keeping the reaction time at 30 min. The FL spectra of a series of CdSe QDs at different molar ratio of TGA/Se are presented in Figure 2. The corresponding samples exhibit emission peaks at about 520 nm. From sample a to c, the emission peak gradually increased with the addition of TGA, while from c to e the emission peak reduced gradually. It turned out that the sample c has a wide full width at half-maximum (FWHM, 50 nm), and the fluorescence intensity of sample c is the strongest. Therefore, the molar ratio of TGA/Se of 2.2 was chosen for further reactions.

**Figure 3:**
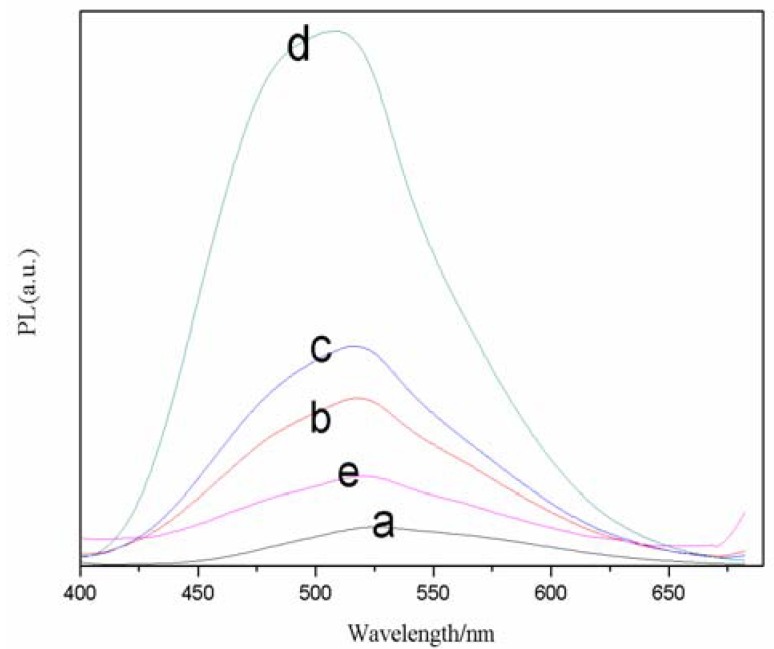
Effects of different molar ratios of Cd/Se on normalized FL spectra of TGA-capped CdSe QDs at freezing temperature. The reaction time was 30 min (a, 1.5; b, 2.5; c, 3.0; d, 3.5; e, 4.0).

The effect of Cd/Se feed molar ratios on the FL spectra of TGA-Capped CdSe QDs is summarized in Figure 3, where the emission peak was collected at 520 nm. From sample a to d, the peak intensity faded with increasing Cd^2+^ concentrations. It was obvious that the full width at half-maximum was more than 95 nm and the best molar radio of Cd/Se was 3.5. Therefore, we chose a TGA/Se molar ratio of 3.5 for future reactions. The reasons for the change can be interpreted that with the increase of Cd molar weight, the concentration of Se precursor compound becomes lower and nucleation is less, so there will be a lot of Cd^2+^ monomers participating in the nanocrystal surface arrangement and reconstruction during the growth of nanocrystal.

**Figure 4:**
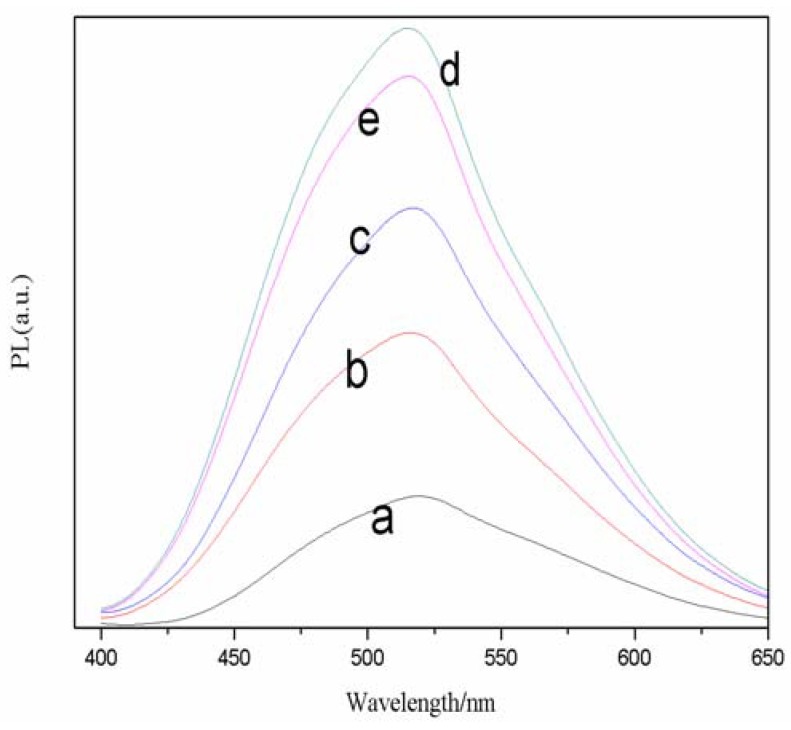
Effects of different pH on normalized FL spectra of TGA-capped CdSe QDs at freezing temperature. Cd/Se/TGA = 3.5:1:2.2. The reaction time was 30 min (a, 8.0; b, 9.0; c, 10.0; d, 10.5; e, 11.0).

As for the pH value, Figure 4 reflects the relationship of fluorescence spectrogram of CdSe QDs with different pH values, such as 8.5, 9.5, 10.0, 10.5 and 11.0. Although the pH value increases, its emission peak still stands around 520 nm. On the other hand, the fluorescence intensity of CdSe QDs changes obviously and it reaches the highest when the pH is 10.5, but a further increase of pH to 11.0 results in a decline in the intensity.

This phenomenon indicates that the pH value may modify the surface structure of the semiconductor nanocrystals. Reaction systems at different pH values will lead to differences in the combination of stabilizer and Cd^2+^, the combination of OH^−^ and Cd^2+^, the reaction of Se^2−^ and Cd^2+^, as well as the size and structure of the QDs, *etc*. For example, the increase of pH value will lead to the formation of a Cd(OH)_2_ shell around the CdSe QDs, and decrease the surface defects, as well as filling the surface cavity of Se^2−^ with OH^−^. Consequently, it compels the electrons’ return to the nuclear eigenstate and eliminates a mass of surface localized states. Elimination of surface dangling bonds brings about an enhanced exciton launch. The CdSe QDs formed by competing reactions have some differences in surface structure. From Figure 4 we also can find that, too strong alkaline conditions (pH = 11) make Se^2−^ defeated in the competition, thus resulting in the reduction of quantum dots, and the decline of fluorescence intensity. The mechanism for the synthesis of CdSe QDs is proposed as follows:


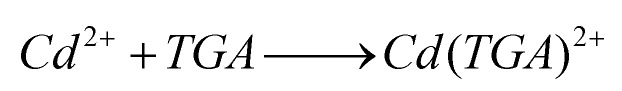



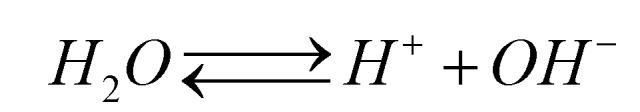



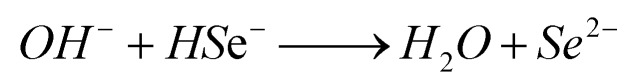



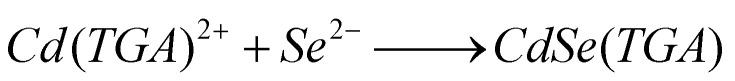



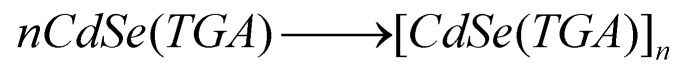


Equation (1) and Equation (4) demonstrate that the slightly basic solution is good for the formation of Cd(TGA)^2+^ complex, while the presence of stronger base leads to the formation of Cd(OH)_2_. Here Cd^2+^, Se^2−^ and TGA instantly formed CdSe (TGA) nuclei as indicated in Equation (7):


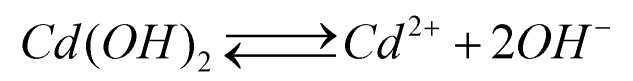



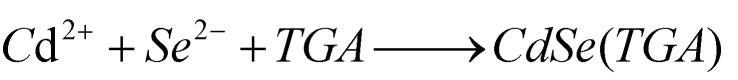


Under the optimum conditions, the prepared CdSe QDs was 14% through the following formula: 


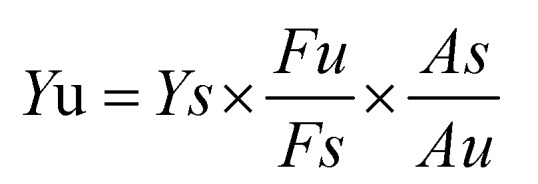


The reason for such high fluorescence quantum yield was that the peak breadth of fluorescence spectrum is so wide that the integral area was very large.

**Figure 5:**
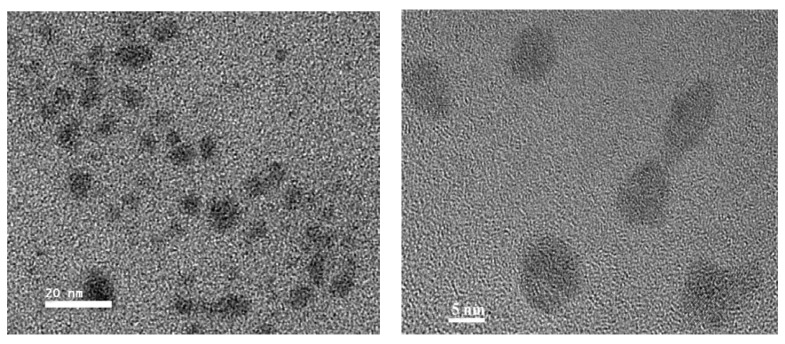
TEM images of synthesized TGA-capped CdSe QDs.

The morphology of the synthesized TGA-capped CdSe QDs was examined by TEM. The data in Figure 5 were collected with the molar ratio of Cd/Se at 3.5 and the reaction time set at 30 min. The typical TEM image of these CdSe QDs showed that QDs was close to spherical, with diameters of about 3–10 nm. The nearly mono-dispersed nanocrystals form long-ranged, well-ordered two dimensional superlattices, which demonstrate that the size and shape of the particles are not very consistent. This result was also in agreement with the fluorescence spectrum.

**Figure 6:**
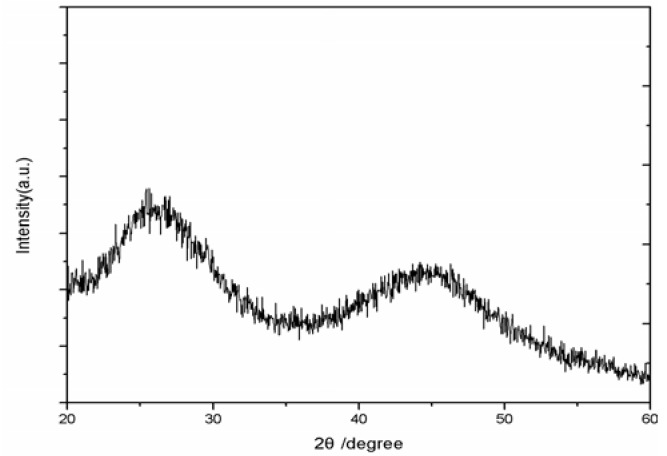
XRD pattern of the CdSe quantum dots synthesized at freezing temperature.

Figure 6 shows the XRD pattern of the CdSe QDs synthesized using the freezing temperature method. The broad peaks imply that the CdSe QDs are very small. The quantum dots belong to cubic phase structure. The main strong peaks are observed are for 111, which clearly matches for CdSe bulk material [16]. No peaks corresponding to impurities were detected, indicating the high purity of the product. The nanocrystals obtained at freezing temperature have less crystallization structure. The completely crystallized structure may not be easy to oxidize due to the compact structure.

## 3 Experimental

### 3.1. Materials and Reagents

All chemicals are obtained commercially available and used without further purification. Se powder and cadmium chloride (CdCl_2_·2.5H_2_O) were purchased from Tianjin Blessed Morning Chemical Reagent Factory. Thioglycolic acid (TGA, Alfa Aesar), and sodium borohydride (NaBH_4_) were purchased from Sinopharm Chemical Reagent Co. Ltd. Deionized water was used.

### 3.2. Characterization of Samples

UV-vis absorption spectra was recorded using a Lambda 950 UV/Vis Spectrophotometer. The photoluminescence and emission spectra were obtained using a LS-55 spectrofluorophotometer (both of them are from Perkin Elmer Instruments Co. Ltd, America). X-Ray power diffractograms (XRD) were recorded using a Philips PW 3040/60 powder diffractometer. Transmission electron microscopy (TEM) images were acquired by using a JEM-2100F transmission electron microscope with an accelerating voltage of 200 KV. The pH value of the system was measured by a PHS-3C pH meter (Shanghai Leici, China). 

### 3.3. Synthesis Process

As shown in Equation (9), to a 25 mL round-bottom flask (I) was added 0.05 mmol Se powder and 0.15 mmol NaBH_4_ and 3.0 mL deionized water, and the reaction was kept at 0 °C (ice bath) for 30 min to give a mixture A. Meanwhile, a mixture of 8.75 mL 0.2 mol/L CdCl_2_ and 1.1 mmol TGA was added to a round-bottom flask (II), then the pH was adjusted to 10.50 with 1 mol/L NaOH, followed by addition of mixutre A via syringe at 0 °C under a nitrogen atmosphere. Finally, the colour of solution became bright yellow. The TGA-capped CdSe QDs were obtained through acetone precipitation with centrifugation at 12,000 rpm for 15 min; these QDs were treated with acetone for three repeated cycles to remove the contaminants.


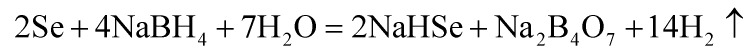


### 3.4. Synthesis Mechanism

It is well-known that inhibiting the particle growth or “Oswald Ripening” can be achieved by the use of capping agents. Usually after the formation of nanoparticles or mixing all of the reactants together, they are capped. The capped-QDs synthesis in this work is different from those of other methods. The metal source (Cd^2+^) was capped prior to introduction of the chalcogenide (Se^2−^). The reaction mechanism can be summarized as follows:


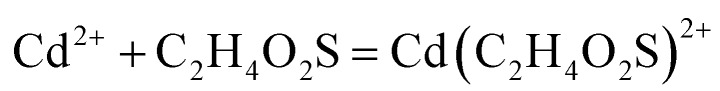



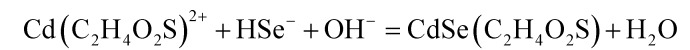



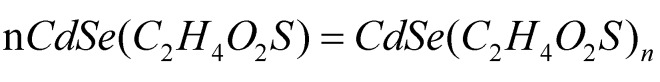


At first, the Cd(C_2_H_4_O_2_S)^2+^ complex is formed by chelating of thioalcohol (C_2_H_4_O_2_S) (Equation (10)), even in the presence of an intense competition from Se^2−^ (Equation (11)). This competition is controlled by the stabilities of the complexes as well as the concentrations of the respective intermediates. CdSe has greater stability, with a Ksp (solubility product constant) value of 6.3 × 10^−36^, than Cd(C_2_H_4_O_2_S)^2+^ complex. Upon titration of Se^2−^, nucleation of TGA-capped CdSe progresses by displacing TGA with Se^2−^. It is energetically favorable for Se^2−^ to incorporate into other Cd(C_2_H_4_O_2_S)^2+^ complexes, rather than continuing to displace TGA in reacted Cd(C_2_H_4_O_2_S)^2+^ complexes to form larger particles. The threshold during nucleation leads to fact that the amount of excess Cd^2+^ will determine the formation of either nanoparticles in solution or bulky species that may precipitate out due to total displacement of the TGA cap. Therefore, with the use of appropriate amounts of capping materials and Cd^2+^, high quality CdSe QDs can be produced. Notably, the amount of Se^2−^ also plays a key role in controlling the size distribution of QDs. The formed CdSe QDs agglomerate as the bulk species is prevented by the steric barrier introduced by the capping group as well as the net negative surface charge of QDs. Finally, the nucleation of the CdSe occurs and forms small particle clusters when the precursors are mixed together at freezing temperature. 

## 4. Conclusions

In conclusion, we utilize a freezing temperature injection technique to obtain CdSe quantum dots with yellow emission. The relatively lower reaction rate allows us to study the dynamics of the crystal growth process and obtain the target products successfully. It is worth noting that the utilization of water as the solvent is more environment friendly compared with the traditional usage of the organic Se sources, such as TOPO-Se, TOP-Se or the toxic organic capping reagents. Thus, this mild and operationally simple route may prove valuable for nanocrystal-related research.
